# Trends in Readmissions Rates and Mortality after Cardiac Resynchronization Therapy in Patients with Nonischemic Cardiomyopathy

**DOI:** 10.2174/011573403X345244250217052010

**Published:** 2025-03-03

**Authors:** Aakash R. Sheth, Harsh P. Patel, Krutharth Pandya, Samarthkumar Thakkar, Kesar Prajapati, Ambica Niar, Mohammad Rafa Labedi, Christopher V. DeSimone, Abhishek Deshmukh

**Affiliations:** 1 Division of Cardiology, University of Pittsburgh Medical Center, Harrisburg, PA 17101, United States;; 2 Division of Cardiology, Southern Illinois University, Springfield, IL 62702, United States;; 3 Department of Medicine, Trumbull Regional Medical Center, Warren, OH 44483, United States;; 4 Division of Cardiology, Houston Methodist Hospital, Houston, TX 77030, United States;; 5 Division of Medicine, Metropolitan Hospital Center, NYC Health+, New York City, NY 10029, United States;; 6 Division of Medicine, Ocean University Medical Center, Brick, NJ 08753, United States;; 7 Department of Cardiovascular Diseases, Mayo Clinic, Rochester, MN 55901, United States

**Keywords:** Nonischemic cardiomyopathy, defibrillator, cardiac resynchronization therapy, heart failure readmission, medication management, cardiac implanted electronic devices (CIED)

## Abstract

**Introduction:**

Advances in cardiac implanted electronic devices (CIED) have significantly improved outcomes for patients with heart failure. However, there is a bereft of recent real-world data on the relative effectiveness of cardiac resynchronization therapy with pacing and defibrillator (CRT-D) and continuous resynchronization therapy with pacing (CRT-P) in patients with nonischemic cardiomyopathy (NICM). We hypothesized that the addition of defibrillation therapy in patients with NICM would offer no significant benefit.

**Methods:**

We searched the National Readmissions Database (NRD) from 2016-2020 to identify hospitalizations with NICM using appropriate ICD-10 diagnosis and procedure codes. The cohort was further divided into groups with NICM and CRT-D implantation and NICM with CRT-P implantation.

**Results and Discussion:**

Our final cohort included 8,801 hospitalizations with NICM and CRT-D implantation and 3,399 hospitalizations with NICM and CRT-P implantation. Propensity matching was performed using comorbidities through multivariate logistic regression. Two thousand nine hundred seventeen hospitalizations were included in each of the two groups, CRT-D and CRT-P. Analysis of the propensity-matched cohorts at 180 days revealed a trend toward lower heart failure readmission, all-cause readmission, and all-cause mortality rates in the group with CRT-P implantation. However, there was no difference noted in the 180-day hazard ratios of HF readmission [1.08 (0.98-1.19); *p* = 0.1], all-cause readmission [1.04 (0.87-1.12); *p* = 0.23], and all-cause mortality [0.83 (0.58-1.19); *p* = 0.32].

**Conclusion:**

It was found that NICM patients with CRT-D have a trend towards higher HF readmissions, all-cause readmission, and all-cause mortality compared to those with CRT-P, but no significant difference was noted in hazard ratios. The findings of our study raise further questions about the need for defibrillator therapy in patients with NICM and merit further studies to better select candidates for each of these therapies.

## 1 INTRODUCTION

Despite improvement in medication management, readmission rates for patients with heart failure remain high, with 1 in 4 patients requiring readmissions within 30 days of discharge and 50% readmission rates in the first 6 months after discharge [1-4]. Advancements in pharmacotherapy and cardiac implanted electronic devices (CIED) have led to significant improvements in the management of heart failure. Cardiac resynchronization therapy (CRT), for example, in appropriately selected patients, has been shown to reduce mortality and hospitalization [5, 6], reverse the cardiac remodeling process, and improve quality of life [7]. However, the effectiveness of CRT with pacing and defibrillator (CRT-D) and CRT with pacing (CRT-P) differs among patients with different etiologies of heart failure, specifically ischemic cardiomyopathy (ICM) and nonischemic cardiomyopathy (NICM). Results of prior studies evaluating the effectiveness of CRT-D in NICM, namely the subgroup analysis of the DANISH trial and the post-hoc analysis of the COMPANION trial, have been conflicting [8, 9]. However, these extensive studies enrolled patients more than a decade ago, and there are limited recent real-world data on this topic. Additionally, most patients with heart failure who have indications for CRT will end up getting the most benefit from resynchronization therapy simply by improvements in their first phase ejection fraction (EF1), which is the ejection fraction (EF) up to the time of maximal ventricular contraction, a measure of early systolic function. This change in EF1 occurs mostly in the first 6 months after implantation of a device and was found to be the strongest predictor of heart failure rehospitalization and death even after a median follow-up of up to 2 years [10]. Hence, in this study, by using the national readmissions database (NRD), we aim to provide a comprehensive comparative analysis of outcomes of CRT-D and CRT-P in patients with NICM and evaluate the readmissions and mortality at six months.

## 2 MATERIALS AND METHODS

### 2.1 Data Source

Data were extracted from the 2016–2020 Nationwide Readmission Database (NRD). NRD is a subset of the Healthcare Cost and Utilization Project (HCUP), sponsored by the Agency for Healthcare Research and Quality. The NRD from 2016–2020 contains data from approximately 17 million discharges across 28 geographically dispersed states, accounting for 60% of the total US resident population and 58.2% of all US hospitalizations [11]. The NRD has been studied and validated in multiple previous studies [12-14]. This study was deemed exempt by the Institutional Review Board as the NRD is a publicly available database that contains deidentified patient information. The data, analytic methods, and study materials are available to other researchers through the HCUP website, https://www.hcup-us.ahrq.gov, and may be used to reproduce the results.

### 2.2 Patient Selection

**Fig. (1):**
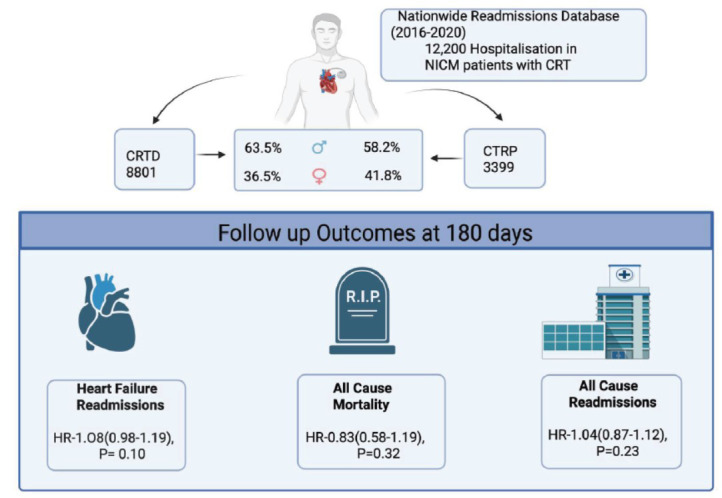
Outcomes of CRT-D *vs.* CRT-P in patients with non-ischemic cardiomyopathy.

We identified hospitalizations with heart failure with reduced ejection fraction using ICD-10 CM codes I50.2 and I50.4 in the primary and secondary diagnosis fields. Out of this population, cohorts with CRT-D and CRT-P were identified using ICD-10 procedure codes 0JH609Z, 0JH639Z, 0JH809Z, 0JH839Z, and 0JH607Z, 0JH637Z, 0JH807Z, and 0JH837Z, respectively. Only hospitalizations with nonischemic cardiomyopathy were analyzed. Those with missing data on age, gender, and mortality were excluded. Hospitalizations with patients aged < 18 years were excluded. The final cohort was divided into NICM with CRT-D (n=9940) and NICM with CRT-P (n=3698) (Fig. **1**).

### 2.3 Variable Ascertainment

We used variables provided in NRD by HCUP to identify baseline characteristics, including age, sex, hospital characteristics, such as bed size, teaching status, and other patient-specific aspects, including median household income category for 'patient's zip code, primary payer, admission type and admission day of the week. We utilized ICD-10-CM codes provided by the Elixhauser Comorbidity Index calculator by HCUP to identify obesity, hypertension, diabetes, chronic obstructive pulmonary disease (COPD), alcohol abuse, tobacco use, peripheral vascular disease, and anemia. Other comorbidities, such as coronary artery disease (CAD), chronic kidney disease stage 3 or more (CKD), atrial fibrillation (AF), liver disease, do not resuscitate status (DNR), and hypothyroidism, were identified using appropriate ICD-10-CM (Table **S1**). Other covariates included demographics, median household income by zip code of residence, payor source/insurance, pertinent hospital factors, such as size, location, teaching status, and variables pertaining to hospitalization, such as length of stay and elective status. Of note, a hospital is regarded as a teaching facility if it is affiliated with an American Medical Association–approved residency program, is a member of the Council of Teaching Teaching Hospitals, has teaching hospitals, or has a full-time equivalent resident-to-patient ratio of ≥ 0.25.

### 2.4 Study Endpoints

The primary endpoints included heart failure readmissions at 180 days. Secondary endpoints included all-cause readmission and all-cause mortality at 180 days.

### 2.5 Statistical Analyses

The categorical variables were compared using the chi-square test, and continuous variables were compared using the student's *t-test*. Propensity matching was performed using comorbidities through multivariate logistic regression. Hospitalizations with similar propensity scores in two groups were matched using a 1-to-1 scheme without replacement using Greedy's method. The maximum propensity score differences (caliper width) of 0.05 were permitted between matched pair observations. Hospitalizations without matched observation were excluded. We used the C-index and Hosmer Lemeshow goodness of fit test to assess the appropriateness of the model. Time-to-event analysis was conducted using Kaplan-Meier curves and Cox-proportional hazard regression. The log-rank test was used to generate *p* values for respective Kaplan-Meier curves. A 2-tailed *p*-value of 0.05 was designated as statistically significant. We adhered to the methodological standard of HCUP. SAS 9.4 (SAS Institute Inc., Cary, North Carolina) and SPSS 26 (IBM Corporation, Chicago, Illinois) were used for statistical analysis.

## 3 RESULTS

We included 12,200 hospitalizations with NICM who had a CRT device implanted. We further divided the cohort into two groups: NICM with CRT-D (n-8801) and CRT-P (n-3399) (Table **1**). In the CRT-D group, 63.5% were males (compared to 58.2% in the CRT-P group). The most common comorbidities were HTN 57.25% in the CRT-D group *vs*. 65.31% in the CRT-P group, COPD 16.45% *vs*. 19.16%, DM 34.11% *vs*. 29.94%, and HLD 44.01% *vs*. 45.96%.

After a one-to-one propensity score matching, 2917 hospitalizations in each cohort were identified. All the comorbidities were balanced between the two groups (Table **1**), except for obesity, which was significantly higher in the CRT-P group.

Analysis of the unmatched cohorts revealed no significant difference between the two groups in the 180-day HF readmission rate [1.01 (0.694-1.08); *p* < 0.771]. Similarly, no difference was found in all-cause readmission at 180 days [1.13 (0.90-1.19); *p* = 0.87] and all-cause mortality rates [1.02(0.94-1.10); *p* = 0.55] (Table **2**).

Analysis of the propensity-matched cohorts revealed lower rates of heart failure readmission, all-cause readmission, and all-cause mortality in the CRT-P group. However, the difference did not reach statistical significance between 180-day hazard ratios of HF readmission [1.08 (0.98-1.19), *p* = 0.1], all-cause readmission [1.04 (0.87-1.12); *p* = 0.23], and all-cause mortality [0.83 (0.58-1.19); *p* = 0.32] (Table **2**).

## 4 DISCUSSION

To our knowledge, this is the first of its kind study from the National Readmission Database comparing the outcomes of CRT-D and CRT-P in hospitalizations with primary diagnoses of NICM. In this study, we report that in the NICM population, the group with CRT-P compared to the one with CRT-D had:

1. No difference in heart failure readmissions at 90 days and 180 days.

2. No difference in all-cause readmissions at either 90 days or 180 days.

3. No difference in all-cause mortality at either 90 days or 180 days.

The evidence of the survival benefit of adding ICD to patients requiring CRT is conflicting. Recent post hoc analysis of the COMPANION trial [9] showed the mortality benefit of using CRT-D in patients with NICM, which contradicts the findings of the DANISH trial [8]. A meta-analysis of 12 clinical trials also showed no difference in survival between the two groups [15]. The ambiguity in evidence on the topic stems from the lack of specific randomized clinical trials comparing the outcomes of CRT-D and CRT-P in patients with NICM. Most existing studies enrolled patients with varied HF etiologies, which further dilutes the findings' power of interest. Additionally, findings of the COMPANION trial [6] were published in 2004, predating several advances in HF management, including drugs, such as angiotensin receptor – neprilysin inhibitor (ARNI) [16], sodium-glucose cotransporter 2 inhibitors [17-20] and improvements in CRT technology, such as using quadripolar leads [21, 22], and updated CRT optimization parameters [23-25]. This significant change in the treatment of heart failure over the last two decades brings into question the relevance of the findings of the COMPANION trial. Further, it highlights the bereft of newer, more prespecified clinical trials for NICM.

The present study shows that the group with CRT-P had trended towards lower heart failure readmission rates at 90 days and 180 days; however, the results did not reach statistical significance. No study in the past has shown a between-device comparison in a NICM population. A post-hoc analysis of the COMPANION trial [26] showed marginally higher rates of HF readmission in the CRT-D group, although the difference was not statistically significant. Similarly, a study on octogenarians showed no difference in HF readmissions between the two device groups [27]. However, both studies included subjects with multiple HF etiologies, including patients with both ICM and NICM. While the exact reason for our findings is unknown, it can be postulated that higher HF readmission rates might be related to inappropriate ICD shocks in patients who might not be candidates for defibrillation therapy. Patients with NICM are classified as being CRT-super responders according to the MADIT-CRT trial [28]. These patients are more likely to achieve normalization or sub-normalization of LVEF, after which they are not considered appropriate candidates for defibrillation therapy [29-31]. These patients might be appropriate candidates for the replacement of CRT-D with CRT-P.

Our study also showed no between-group difference in all-cause readmissions. Data on this topic conflict with some studies [26, 32], showing no difference in rates of hospitalizations between CRT-D and CRT-P groups. In contrast, other studies show higher rates of hospitalizations in the ICD group due to direct myocardial injury, contraction band necrosis, fibrosis, and possible persistent inflammation resulting from spontaneous ICD shocks, leading to increased hospitalizations [33]. In claims data from fee-for-service Medicare beneficiaries, CRT-P patients had a lower probability of hospitalizations [34]. Given that in our study, the HF readmission rates were lower in the CRT-P group, albeit not statistically significant, it can be safely assumed that the CRT-P group had higher non-HF-related admissions compared to the CRT-D group. However, given that these findings were also persistent after propensity matching further highlights the bereft of granular information in NRD. This further implicates the need for a specific randomized comparison of these treatment strategies.

Lastly, our study showed no difference in mortality between the two groups. These findings further strengthen the results of the DANISH trial, which is by far the most contemporary RCT comparing CRT-D and CRT-P in NICM patients, and contradict the findings of the post-hoc analysis of the COMPANION trial. It is well known that the mortality benefit of a defibrillator is mainly derived from its prevention of sudden cardiac death. Hence, one possible explanation for our findings is that the NICM group is less prone to sudden cardiac death than the ICM group, causing attenuation of the mortality benefit of ICD. Additionally, as explained above, the benefits of CRT-D might be negated due to reverse cardiac remodeling and improvement in LVEF by more than 35% in patients with NICM with prolonged CRT therapy.

## 5 LIMITATIONS

First, we used NRD, an administrative database derived from ICD-9 and ICD-10 billing codes. This makes the data inherently prone to coding errors, potentially limiting the accuracy of our results. Also lacking are the details of specific pre-procedural characteristics, such as baseline echocardiographic findings, frailty, and functional status of patients; procedural characteristics, such as the make and models of CRT; and post-procedural characteristics, such as post-procedure echocardiographic findings, intensive care stay, all of which could greatly impact the results. This dataset also lacks information regarding patients' medication treatment regimens, which are important and essential predictors of readmissions in patients with heart failure. Additionally, the database provides no mention of the specific causes of death for each admission, especially about what percentage of patients had sudden cardiac death. There is also no information regarding practitioner expertise, which could impact the success rates of the procedures and readmission rates. We used a robust propensity score matching to adjust for all measurable cofounders. However, it still does not account for unmeasured confounders. Lastly, this study only reports results up to 6 months of follow-up. Despite these limitations, a strength of the study is the large, nationally representative sample size, which could negate more nuanced limitations of other small, single-center studies on the same topic.

## CONCLUSION

In conclusion, the findings of this study raise further questions about the relevance of defibrillator therapy in patients with NICM. While CRT-P therapy is cost-effective and potentially avoids overtreatment, a significant drawback is that if defibrillator therapy is needed in the future, the patient would have to be re-operated. Hence, we need robust models predicting the impact of HF interventions with reasonable accuracy (85-90%). This can be started by incorporating variables, such as C-reactive protein to albumin ratio [35] or prognostic nutritional index [36], into our decision to implant ICD in patients with heart failure. Regardless, more randomized clinical trials comparing the efficacy of CRT-P and CRT-D in NICM patients are also needed. The present study can hopefully serve as a nidus for more extensive prospective studies.

## Figures and Tables

**Table 1:** Baseline characteristics of patients with NICM undergoing CRTD *vs*. CRTP.

-	**Unmatched**			**Propensity Matched**		
	**CRTD (n=8801)**	**CRTP (n=3399)**	***p*-value**	**CRTD (n=2917)**	**CRTP (n=2917)**	***p*-value**
**Age group**	-	-	<0.0001	-	-	0.02
18-48 years	12.07%	4.17%	-	3.76%	4.66%	-
49-64 years	31.87%	15.82%	-	16.31%	17.82%	-
65-74 years	28.63%	24.72%	-	30.47%	27.51%	-
≥ 75 years	27.42%	55.30%	-	49.46%	50.01%	-
**Sex**	-	-	<0.0001	-	-	0.01
Male	60.51%	54.10%	-	58.06%	54.97%	-
Female	39.49%	45.90%	-	41.94%	45.03%	-
**Median household income (quartile range)**	-	-	<0.0001	-	-	0.45
0-25th	30.08%	26.35%	-	27.84%	27.39%	-
25th-50th	28.10%	27.67%	-	28.58%	27.24%	-
50th-75th	23.96%	25.68%	-	24.59%	24.97%	-
75th-100th	17.86%	20.30%	-	18.99%	20.40%	-
**Primary payer**	-	-	<0.0001	-	-	0.89
Medicare	71.93%	83.99%	-	82.72%	82.59%	-
Medicaid	28.07%	16.01%	-	17.28%	17.41%	-
**Type of admission**	-	-	<0.0001	-	-	<0.0001
Elective	25.29%	21.12%	-	27.70%	22.29%	-
Non-elective	74.71%	78.88%	-	72.30%	77.71%	-
**Hospital characteristics**	-	-	-	-	-	-
**Hospital bed-size**	-	-	0.16	-	-	0.92
Small	30.22%	31.50%	-	30.30%	30.41%	-
Large	69.78%	68.50%	-	69.70%	69.59%	-
**Hospital location and teaching status**	-	-	0.77	-	-	0.0001
Rural	18.06%	18.28%	-	21.84%	17.89%	-
Urban teaching	81.94%	81.72%	-	78.16%	82.11%	-
**Co-morbidities**	-	-	-	-	-	-
OSA	15.35%	14.55%	0.27	13.44%	14.48%	0.24
Tobacco use	10.47%	7.51%	<0.0001	7.97%	7.59%	0.58
Hypertension	57.25%	65.31%	<0.0001	69.49%	64.09%	0.32
Diabetes mellitus	34.11%	29.94%	<0.0001	29.54%	30.95%	0.23
COPD	16.45%	19.16%	0.0004	18.23%	18.86%	0.53
Hypothyroidism	13.66%	17.46%	<0.0001	16.75%	15.38%	0.15
Hyperthyroidism	0.87%	0.92%	0.78	0.59%	1.00%	0.07
Obesity	22.21%	19.10%	0.0002	17.59%	19.81%	0.02
Alcohol abuse	4.69%	3.31%	0.0008	3.51%	3.52%	0.97
PVD	4.45%	4.84%	0.35	3.57%	4.85%	0.20
Anemia	21.39%	27.30%	<0.0001	25.09%	25.43%	0.75
Liver disease	3.50%	3.20%	0.41	2.86%	3.27%	0.36
Hyperlipidemia	44.01%	45.96%	0.05	45.87%	46.18%	0.81
**In-hospital mortality**	1.21%	1.61%	0.08	1.47%	1.39%	0.84
**Disposition**	-	-	<0.0001	-	-	0.0004
Routine	66.55%	51.41%	-	60.11%	54.21%	-
Short term hospital	0.36%	0.40%	-	0.25%	0.40%	-
Another facility	12.33%	21.97%	-	16.84%	19.58%	-
Home health care	19.23%	24.37%	-	21.11%	24.19%	-

**Table 2:** Unmatched and propensity-matched outcomes.

-	**Unmatched**			**Propensity Matched**		
	**CRTD (n=8801)**	**CRTP (n=3399)**	***p*-value**	**CRTD (n=2917)**	**CRTP (n=2917)**	***p*-value**
Heart failure readmission	14.11%	10.13%	<0.0001	14.36%	9.97%	<0.0001
Hazard ratio	1.01 (0.94-1.08)		0.77	1.08 (0.98-1.19)		0.1
All-cause readmission	32.14%	30.89%	0.18	33.72%	30.25%	0.004
Hazard ratio	1.13 (0.90-1.19)		0.87	1.04 (0.87-1.12)		0.23
Readmission mortality	3.14%	2.66%	0.15	3.05%	2.56%	0.25
Hazard ratio	1.02 (0.94-1.10)		0.55	0.83 (0.58-1.19)		0.32

## Data Availability

The data, analytic methods, and study materials are available to other researchers through the HCUP website, https://www.hcup-us.ahrq.gov, and may be used to reproduce the results.

## LIST OF ABBREVIATIONS

AF: Atrial Fibrillation
CAD: Coronary Artery Disease
CIED: Cardiac Implanted Electronic Devices
CKD: Chronic Kidney Disease Stage 3 or more
COPD: Chronic Obstructive Pulmonary Disease
CRT-D: Cardiac Resynchronization Therapy with Pacing and Defibrillator
CRT-P: Continuous Resynchronization Therapy with Pacing
HCUP: Healthcare Cost and Utilization Project
NICM: Nonischemic Cardiomyopathy
NRD: National Readmissions Database

## STANDARDS OF REPORTING

STROBE guidelines were followed.
