# Supplementary material for: Trends in Readmissions Rates and Mortality after Cardiac Resynchronization Therapy in Patients with Nonischemic Cardiomyopathy
Source: Curr Cardiol Rev. 2025 Mar 3;21(6):e1573403X345244. doi: 10.2174/011573403X345244250217052010 (PMC12676016; doi:10.2174/011573403X345244250217052010)
Supplement: Supplementary file 1 [file CCR-21-6-E1573403X345244_SD1.pdf]

Supplementary Material

Trends in Readmissions Rates and Mortality after Cardiac Resynchronization Therapy in Patients with Nonischemic Cardiomyopathy

Aakash R. Sheth[1,*], Harsh P. Patel[2], Krutharth Pandya[3], Samarthkumar Thakkar[4], Kesar Prajapati[5], Ambica Niar[6], Mohammad Rafa Labedi[2], Christopher V. DeSimone[7] and Abhishek Deshmukh[7,*]

[1]*Division of Cardiology, University of Pittsburgh Medical Center, Harrisburg, PA 17101, United States;* [2]*Division of Cardiology, Southern Illinois University, Springfield, IL 62702, United States;* [3]*Department of Medicine, Trumbull Regional Medical Center, Warren, OH 44483, United States;* [4]*Division of Cardiology, Houston Methodist Hospital, Houston, TX 77030, United States;* [5]*Division of Medicine, Metropolitan Hospital Center, NYC Health+, New York City, NY 10029, United States;* [6]*Division of Medicine, Ocean University Medical Center, Brick, NJ 08753, United States;* [7]*Department of Cardiovascular Diseases, Mayo Clinic, Rochester, MN 55901, United States*

Supplemental Table 1: ICD 10 codes for respective variables.

	ICD 10 codes
Heart failure with reduced ejection fraction	I50.2, I50.4
CRTD (procedural code)	0JH609Z,0JH639Z,0JH809Z,0JH839Z
CRTP (procedural code)	0JH607Z,0JH637Z,0JH807Z,0JH837Z
Comorbidities	
OSA	G47.33
Obesity	E66, Z68.3, Z68.4
Hypertension	I10, I11, I12, I13, I14, I15, I16
Diabetes	E08, E09, E10, E11, E13
Tobacco use	F17
COPD	J41, J42, J43, J44
CKD stage 3 or more	N18.3, N18.4, N18.5, N18.6, E08.2, E09.2, E10.2, E11.2, E13.2, I12, I13
Hyp0thyroidism	E00,E01,E02,E03,E04
Alcohol Disorder	F10, Z71.40, K29.2, G31.2, K85.2, K86.0, T51, I42.6, K70, G62.1
Peripheral vascular disease	E08.5, E09.5, E10.5, E11.5, E13.5, I73, T82.856, Z98.62, Z95.820
Anemia	D5x, D60, D61, D62, D63, D64, D46.0, D46.1, D46.2, D46.4, O99.0
Heart failure readmission	I11.0, I13.0, I13.2, I50
All the ICD 10 codes were verified by 2 independent authors (SA and CJ) using ICD10data.com website.	
